# Spindle Bursts in Neonatal Rat Cerebral Cortex

**DOI:** 10.1155/2016/3467832

**Published:** 2016-01-13

**Authors:** Jenq-Wei Yang, Vicente Reyes-Puerta, Werner Kilb, Heiko J. Luhmann

**Affiliations:** Institute of Physiology, University Medical Center of the Johannes Gutenberg University, Duesbergweg 6, 55128 Mainz, Germany

## Abstract

Spontaneous and sensory evoked spindle bursts represent a functional hallmark of the developing cerebral cortex in vitro and in vivo. They have been observed in various neocortical areas of numerous species, including newborn rodents and preterm human infants. Spindle bursts are generated in complex neocortical-subcortical circuits involving in many cases the participation of motor brain regions. Together with early gamma oscillations, spindle bursts synchronize the activity of a local neuronal network organized in a cortical column. Disturbances in spindle burst activity during corticogenesis may contribute to disorders in cortical architecture and in the activity-dependent control of programmed cell death. In this review we discuss (i) the functional properties of spindle bursts, (ii) the mechanisms underlying their generation, (iii) the synchronous patterns and cortical networks associated with spindle bursts, and (iv) the physiological and pathophysiological role of spindle bursts during early cortical development.

## 1. Introduction

Network-driven spindle-like oscillations are a functional hallmark of the developing cerebral cortex. During late prenatal and early postnatal stages of development, spontaneous spindle-like oscillations have been identified as physiological activity patterns in various neocortical areas of different mammalian species [1–4]. In humans, so-called “delta brushes” can be observed in EEG recordings from preterm babies already at gestational week 28, that is ~12 weeks before normal birth of a full-term neonate (for review see [5]). In AC-filtered EEG recordings, delta brushes are brief rhythmic delta waves (0.3–1.5 Hz) of 50–300 *μ*V amplitude with a superimposed burst of fast rhythm (>8 Hz, the “brush”). It has been suggested that delta brushes in human preterm infants correlate with so-called spindle bursts recorded in rodents during the early postnatal period [6]. From a developmental point of view, these early spontaneous activity patterns in developing rodent and human cerebral cortex are probably of similar or even identical origin. Rats and mice are altricial-born in a far less mature condition than humans. In rodents the degree of neocortical development at the day of birth (postnatal day [P] 0) can be compared to the stage of human cortex between gestational weeks 28 and 32 [7] and the cerebral cortex of a P12–P14 rat is comparable to that of the full-term newborn human baby [6, 8]. In preterm infants born between gestational weeks 28 and 32, Milh et al. [9] and Colonnese et al. [10] recorded EEG signals containing spontaneous and stimulus-evoked delta brushes with oscillatory activity in the frequency range of 8 to 25 Hz, suggesting that spontaneous delta brushes may represent a physiological neocortical activity pattern of the human fetus in utero.

**Figure 1:**
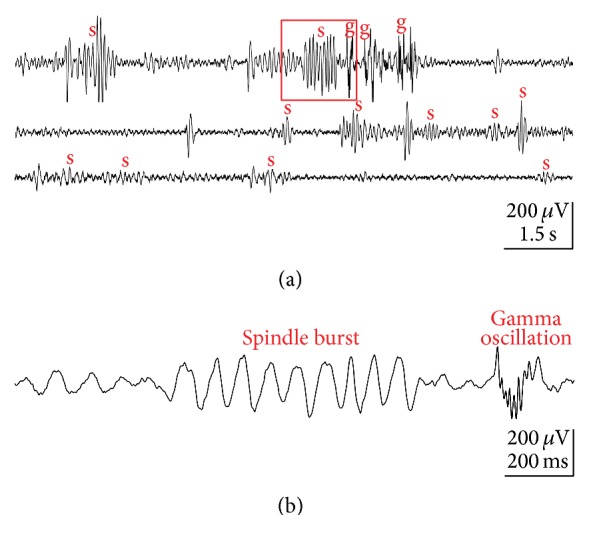
Spontaneous network activity recorded with an extracellular electrode array in primary somatosensory cortex of a P3 rat under light urethane anesthesia. (a) Continuous field potential (FP) recording showing several spindle bursts (s) and gamma oscillations (g). (b) Example of spindle burst and gamma oscillation marked in (a) by red box and displayed at expanded timescale. Reproduced with permission from [17].

In the cerebral cortex of rodents, spindle bursts (beside short gamma oscillations) constitute the majority of spontaneous activity during the first postnatal week (Figure 1). These spindle bursts resemble in their appearance spindles recorded in the adult brain during sleep. These sleep spindles are one of the hallmarks of human stage 2 sleep—for comprehensive overviews the reader is referred to recent reviews by Lüthi [11] and by McCormick et al. [12]. However, in humans sleep spindles appear 4 to 9 weeks after birth, which is much later than the disappearance of delta brushes around the end of the first postnatal week [13], thus excluding the hypothesis that delta brushes or spindle bursts gradually develop into sleep spindles. In addition, the frequency profile of spindle bursts and delta brushes displays a rather broad frequency distribution up to 25 Hz [9], whereas sleep spindles present oscillations in a narrower band of ~10–15 Hz [11].

The present review focuses on spindle burst activity in the cerebral cortex of the developing rat during the first postnatal week and summarizes our current understanding (i) on the functional properties of spindle bursts, (ii) the mechanisms underlying their generation, (iii) the synchronous patterns and cerebral networks associated with spindle bursts, and (iv) the physiological and pathophysiological role of spindle bursts during early cortical development.

## 2. Functional Properties of Spindle Bursts in Neonatal Rat Cerebral Cortex 

**Figure 2:**
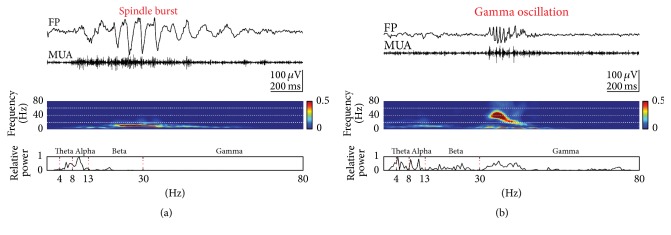
Properties of spontaneous spindle bursts and gamma oscillations. (a) Spindle burst recorded in barrel cortex of a P1 rat (top) and corresponding MUA after 200 Hz high-pass filtering (below). Color-coded frequency plot shows the wavelet spectrum of the field potential recording at identical timescale. Fast Fourier Transformation (FFT) of the field potential recording illustrating the relative power of the displayed spindle burst with maximal power at 10 Hz frequency (bottom). (b) Gamma oscillation (top) recorded in barrel cortex of a P3 rat and corresponding MUA (below). Wavelet and FFT spectrum reveal prominent gamma activity between 30 and 50 Hz. Reproduced with permission from [17].

Two distinct activity patterns can be observed in the neonatal rat cerebral cortex in vivo: gamma oscillations and spindles bursts (Figures 1 and 2) [14–19]. Gamma oscillations have a duration of 100 to 300 ms, a frequency of 30 to 40 Hz and appear spontaneously every 10 to 30 s (Figures 1 and 2(b)) [17]. The properties and functional role of gamma oscillations as well as the mechanisms underlying their generation have been recently reviewed by Khazipov and coworkers [20]. Spindle bursts are characterized by their spindle-like oscillatory appearance, have a duration of 0.5 to 3 s and a frequency in the range of 8 to 30 Hz, and occur spontaneously every ~10 s (Figures 1 and 2(a)). As recognized in full-band direct-current (DC) coupled recordings, spindle bursts are nested in slow (delta) waves, which in infant rats [21], and preterm human babies [22, 23] have been termed “spontaneous activity transients” (SATs). Spindle bursts have been described in primary somatosensory cortex (S1) including barrel cortex [14, 16–19], in primary visual cortex (V1) [10, 15], in primary motor cortex (M1) [19], and in prefrontal cortex [24] of anesthetized and awake rats during the first postnatal week. In S1, spontaneous and stimulus-evoked spindle bursts can be observed as early as P0 [17, 18]. In rats, the incidence of spindle bursts declines during the second postnatal week and sporadic spindle bursts are obscured by the ongoing neocortical activity. Likewise in humans, delta brushes are replaced by more continuous EEG activity at birth of a full-term infant. This gradual developmental shift from a highly synchronized state of spontaneous activity to a desynchronized state seems to be a fundamental network property of the cerebral cortex during early neonatal stages.

**Figure 3:**
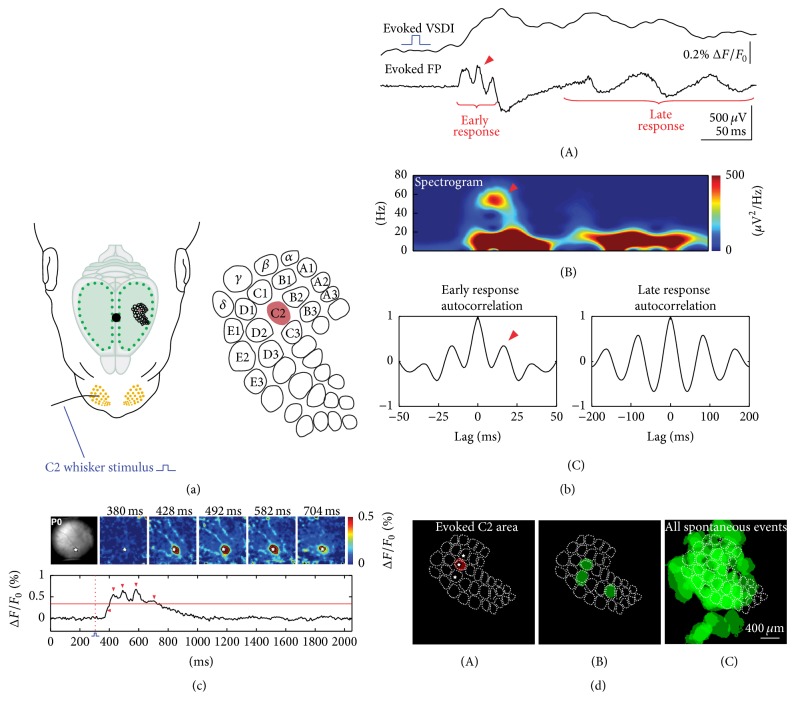
Stimulus-evoked (a–c) and spontaneous (d) network activity recorded in barrel cortex of newborn rats in vivo. (a) Schematic illustration of the experimental setup for selective mechanical stimulation of a single whisker (here whisker C2) (left). Black dot indicates bregma position. Schematic illustration of the barrel field with localization of the C2 barrel (red area) as revealed by stimulation of different single whiskers and monitoring the resulting VSDI response (right). (b) Cortical VSDI and simultaneous FP response to single whisker stimulation in a P1 rat. Note presence of early gamma oscillation followed by late spindle burst in the FP response (A) with typical frequency distribution in the corresponding spectrogram plot (B) and autocorrelograms (C). (c) VSDI in the barrel cortex of a P0 rat following C2 whisker stimulation at the time point of 300 ms (red-dotted line). The localization of the C2-whisker representation in the barrel cortex and 5 successive poststimulus VSDI responses are shown in the upper rows. White stars indicate the center of the C2-whisker-evoked response. Lower rows show 2 s long optical recordings in which the time points of the 5 successive frames are marked by red arrow heads. Red horizontal line indicates the half-maximal response amplitude. Note that the evoked activity is restricted to the C2 barrel and does not propagate to neighbouring columns as in slightly older animals (for more information see [18]). (d) Spontaneous gamma-spindle activity synchronizes early cortical columns. Single whisker stimulation-induced VSDI response (here C2 whisker) recorded in the barrel cortex of a P1 rat (A). The barrel field map was generated on the basis of a cytochrome oxidase stained horizontal section and aligned according to the evoked VSDI responses to single whisker stimulation. White star marks the center of the activated barrel-related columns B2, C2, and D2. Three single spontaneous events localized in a single (pre-) barrel-related column (B) and overlay of all spontaneous events ((C), *n* = 75) recorded in this P1 rat. Note complete coverage of the whole barrel field map by local spontaneous oscillations. Reproduced with permission from [18].

Spindle bursts synchronize the activity of a local neuronal network of 200 to 400 *μ*m in diameter, which resembles the dimension of a whisker-related neocortical column in the immature barrel cortex (Figure 3) [18, 25]. Using a combination of voltage-sensitive dye imaging and high-density multielectrode recordings in P0-P1 rat barrel cortex in vivo, Yang et al. could demonstrate that this early spontaneous activity constitutes the later emergence of the whisker-related barrel field map. These data indicate that spontaneous activity patterns at this early age form “functional precolumns,” supporting the concept of the existence of “ontogenetic columns” in the radial unit hypothesis [26]. Further support for this hypothesis comes from in vivo two-photon calcium imaging in the barrel cortex of both anesthetized and nonanesthetized newborn mice, demonstrating highly synchronous spontaneous burst activity, reminiscent of spindle bursts, in local networks of 100 to 200 *μ*m in diameter [27]. These data strongly indicate that early spindle bursts, and probably also gamma oscillations (for review [20]), synchronize early neocortical networks into functional columns at a developmental stage when the upper layers 2/3 have not even been formed (i.e., in rats at P0). At this developmental stage, thalamocortical afferents have not reached layer 4 and instead transiently innervate the subplate (for review [28, 29]). Thalamic afferents form transient glutamatergic synapses with surprisingly mature properties including AMPA and NMDA receptors [30–32]. In vitro studies in acute brain slice preparations and in intact whole cortical hemisphere preparations [33] have demonstrated that oscillatory network activity in the frequency range of spindle bursts depend on an intact subplate [34, 35]. Selective removal of the subplate in S1 in vivo causes a significant decline in the occurrence of spontaneous spindle bursts and disturbances in the development of the cortical architecture in the barrel cortex [36]. These data further support the hypothesis that spindle bursts in developing cerebral cortex fulfill an important role in the maturation of the neocortical architecture. In the next section we will discuss the network and molecular mechanisms underlying the generation of spindle bursts in neonatal cerebral cortex.

## 3. Generation of Spindle Burst Activity

The rodent cerebral cortex develops rapidly during late prenatal and early postnatal stages and at least four different activity patterns may occur sequentially between birth and the end of the first postnatal week (for review [37]). Furthermore, the cortex shows a mediolateral and anterior-posterior gradient in development and within the same neocortical area neurons in upper layers 2/3 are ~2 days younger compared to lower cortical layers. Therefore, it is most important to compare identical ages and identical brain regions. Many inconsistencies in the literature on the properties of spontaneous activity patterns in newborn rodents and their underlying mechanisms can be explained by the fact that these important developmental differences are often ignored. In addition, 2 days in early rodent cortical development make a large difference, so that the neocortex of a P0 rat (without layer 2/3) cannot be compared to that of a P2 rat (with almost complete lamination).

Already in newborn (P0-P1) rat barrel cortex in vivo, mechanical stimulation of a single whisker elicits in field potential recordings and to some extent detectable also in voltage-sensitive dye imaging (VSDI) a sequence of an early gamma oscillation followed by a spindle burst (Figure 3(b)) [18]. At this age, the thalamocortical activity reaches the developing cortical network largely via the subplate [17, 31, 32] and is amplified by an intrinsic gap-junction coupled network within the subplate and cortical plate [28, 34]. Spontaneous and evoked delta brushes can be observed in premature human neonates of 28–32 weeks postconceptional age [9], a developmental stage when the human cerebral cortex resembles that of a newborn rat. Impressive examples of large delta brushes are provided in the supplementary EEG videos of Milh et al. [9], demonstrating that a single touch elicits a large oscillatory response in the somatosensory evoked potential (SEP) recorded above the contralateral parietal cortex. In mature human cortex, SEPs with smaller amplitudes and shorter durations can be only obtained after averaging of at least 100 epochs. Thus, in both species, rats and humans, at a comparable stage of cortical development mechanical stimulation of the sensory periphery elicits in S1 spindle bursts and delta brushes, respectively.

**Figure 4:**
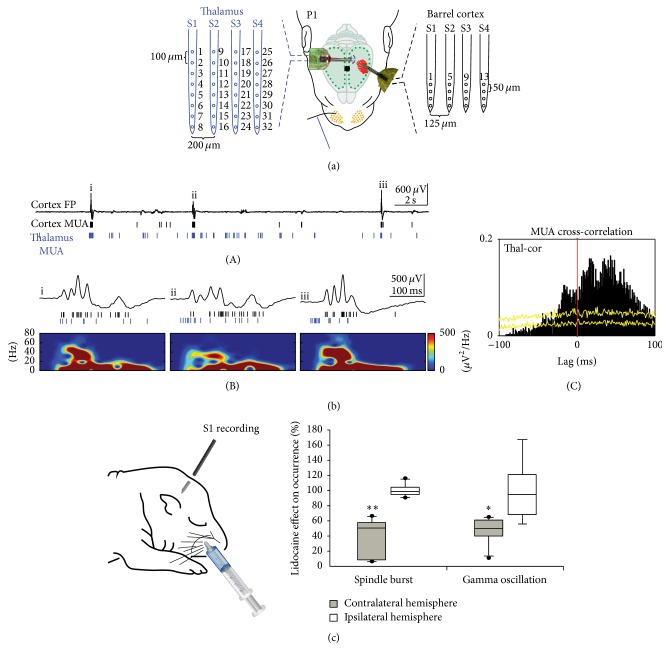
Subcortical origin of spontaneous spindle bursts and gamma oscillations. (a) Schematic illustration of the experimental setup for simultaneous multichannel recordings of sensory evoked and spontaneous activity in both the thalamus and barrel cortex of a newborn rat. A 4-shank 32-channel electrode is located in the ventral posteromedial nucleus (VPM) of the thalamus (blue) and a 4-shank 16-channel electrode in the cortex (black). (b) Simultaneous 40 s recording of spontaneously occurring activity in the barrel cortex and VPM thalamus of a P1 rat. Upper trace represents the cortical FP recording; cortical (black bars) and thalamic MUA (blue bars) are presented below (A). The 3 spontaneous events i–iii marked in (A) are shown at higher resolution with corresponding spectrograms (B). Cross-correlogram of the spontaneous multiunit activity recorded simultaneously in the thalamus and barrel cortex (C). Yellow lines represent results from the shuffled dataset (for details see [18]). (c) Inactivation of the sensory periphery by injection of 2% lidocaine into the whisker pad (left) causes a significant reduction in the relative occurrence of spindle bursts and gamma oscillations recorded in the contralateral barrel cortex of 6 newborn rats (filled bars), whereas both activity patterns in the ipsilateral cortex are not affected (open bars). Data are expressed as box plots, and asterisks mark significant differences ^*∗*^ *P* < 0.05 and ^*∗∗*^ *P* < 0.01. Reproduced with permission from [18] (a, b) and from [17] (c).

Simultaneous multielectrode recordings in the barrel cortex and in the ventral posteromedial nucleus (VPM) of the somatosensory thalamus of P0-P1 rats in vivo have demonstrated that the majority of spontaneous cortical spindle bursts and also gamma oscillations are not generated within S1, but rather in subcortical structures or outside of S1 (Figures 4(a) and 4(b)) [18]. At this age a local, functionally defined lesion in the VPM blocks the whisker stimulation-induced cortical responses and also profoundly reduces the spontaneously occurring cortical burst activity [18], further demonstrating that the majority of the spontaneous spindle bursts in P0-P1 rat barrel cortex are generated in subcortical structures of the whisker-to-barrel cortex pathway (for further information see [25]). Silencing the sensory periphery by injection of lidocaine into the whisker pad causes a significant reduction in the occurrence of spontaneous spindle bursts and gamma oscillations by ~50% (Figure 4(c)) [17], indicating that during this developmental period at least half of the spontaneous burst activity in S1 is related to activity in the sensory periphery. A similar peripheral generation of spontaneous burst activity may occur in human preterms and fetuses between gestational weeks 28 and 32 [9, 22].

Similar to the spindle bursts in S1, spontaneous spindle bursts in V1 are also largely generated in the sensory periphery. Spontaneous retinal activity, so-called “retinal waves” (for review [38]), provide the primary drive for spindle bursts in newborn rat V1, as demonstrated by simultaneous recordings from the retina and V1 [15]. Intraocular injection of forskolin, which augmented retinal waves, increased the occurrence of V1 spindle bursts, and removal of the retina reduced the spindle bursts frequency [15]. As in the somatosensory system, spindle bursts in V1 can be also evoked by stimulation of the sensory periphery. However, since rod- and cone-mediated visual signaling is not functional in rats during the first postnatal week, spindle bursts cannot be evoked by light flashes before P8 [10]. At that age, the neocortical response in V1 consists of an early visual evoked response followed by an evoked spindle burst. Similar responses could be observed in V1 of preterm infants once photoreceptor mediated light responses occur in the retina [10] (for review [6]).

**Figure 5:**
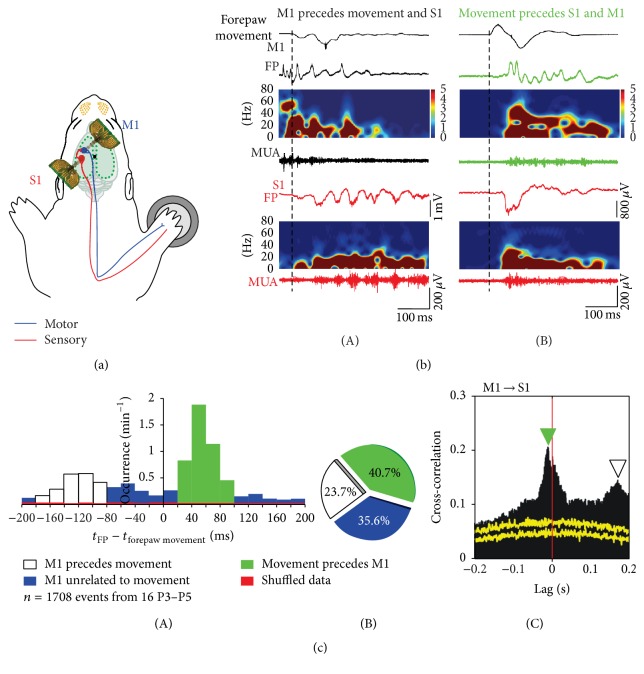
Movement-correlated synchrony of spontaneous network activity in S1 and primary motor cortex (M1) in newborn rat in vivo. (a) Schematic illustration of the experimental setup with multielectrode arrays in S1 (red) and M1 (blue). Piezo element attached to the contralateral forepaw monitors movements. Blue line indicates the motor pathway and red line the sensory pathway. (b) Relationship between forepaw movements and cortical activity in S1 and M1 in a P4 rat. (A) Spontaneous activity in M1 (black) elicited forepaw movement and preceded spindle burst in S1 (red). Black dashed line indicates time point of forepaw movement. Top black trace shows forepaw movement. (B) Spontaneous forepaw movement preceded activity in M1 (green) and spindle burst in S1 (red). (c) Relationship between spontaneous activity in M1 and S1 and forepaw movements. (A) Bar diagram illustrating the occurrence of FP activity, which preceded forepaw movements (blank box), followed forepaw movements (green), and were unrelated to movement (blue) in 16 P3–P5 rats. Red bars represent results from the shuffled dataset. (B) Pie diagram showing the percentages of the three patterns (1708 events from 16 P3–P5 rats during 10 min unstimulated recordings). (C) Cross-correlation of MUA between S1 and M1. Note that S1 MUA precedes M1 MUA (green arrowhead) and M1 MUA precedes S1 MUA (blank arrowhead). Yellow traces represent results from the shuffled dataset. Reproduced with permission from [19].

Whereas a large amount of experimental data has shown that retinal waves provide the main trigger for the cortical V1 spindles bursts, it is not completely understood which pacemaker drives the spontaneous activity in S1 and how spontaneous activity is generated in the somatosensory periphery. In P3–P6 rats spontaneous whisker movements occur during active sleep and are correlated with activation of whisker-related cortical columns in the barrel cortex [39]. In newborn rats the proprioceptive feedback from self-generated myoclonic movements trigger spindle bursts in S1 (for review [40]). Spontaneous limb movements of the human fetus during the third trimester of gestation, or those of the preterm infant associated with delta brushes in S1, are similar to these twitching movements of the neonatal rat and can be also triggered by sensory feedback [9]. Kreider and Blumberg have demonstrated in 1-week-old rats that the mesopontine region plays a central role in the generation of myoclonic twitching [41]. Khazipov et al. have shown in newborn rats that spatially confined spindle bursts in S1 are triggered in a somatotopic manner by sensory feedback signals from spontaneous muscle twitches [14]. These spontaneous movements are generated by neuronal networks in the spinal cord, but spindle bursts persisted at a reduced frequency after sensory deafferentation (spinal cord transection) in S1 [14], indicating that spindle burst activity can be also generated in neocortical or thalamocortical circuits during this early period of development. This assumption is also supported by the observation that silencing of the sensory periphery causes only a ~50% reduction in the occurrence of spontaneous spindle bursts (Figure 4(c)). However, since it cannot be excluded that the remaining portion of spindle bursts actually conveys activity from adjacent or distant sensory areas (transmitted via inter- and intrahemispheric connections, see next chapter), the outcome of this experiment may underestimate the contribution of the sensory periphery. Simultaneous monitoring of forepaw movements, VSDI, and extracellular multielectrode recordings in S1 and M1 of P3–P5 rats under light urethane anesthesia have demonstrated that tactile forepaw stimulation triggers spindle bursts in S1, followed by gamma and spindle bursts in M1 (Figure 5) [19]. Focal electrical stimulation of corticospinal tract neurons in layer 5 of M1 mimicking physiologically relevant 40 Hz gamma or 10 Hz spindle burst activity reliably elicited forepaw movements, indicating that M1 cortical spindle bursts are capable of triggering muscle twitches at this age. However, only 23.7% of the spontaneous bursts in M1 triggered forepaw movements and were followed by spindle bursts in S1 (Figures 5(b)(A) and 5(c)), indicating that only a fraction of M1 activity transients triggers motor responses directly. In 40.7% of the cases, spontaneous movements preceded the burst activity in M1 and S1 (Figures 5(b)(B) and 5(c)), suggesting that this activity may arise from subcortical regions in the brainstem or spinal cord. The remaining 35.6% of the M1 bursts were unrelated to any movements (Figure 5(c)). The finding that 23.7% of the movements were triggered by M1 bursts as observed by An et al. [19] is in contrast to previous observations, which demonstrated that dissection of neocortical inputs fails to suppress muscle twitches in rat pups [41].

In summary, these data indicate that neocortical spindle bursts in newborn rodents (and delta brushes in human fetus during the third trimester or in preterms) are generated by central pattern generator (CPG) circuits in spinal cord, brainstem, and motor cortex (for CPG circuits in mature brain see [42–44]).

Neuropharmacological studies provided insights into the molecular mechanisms underlying the generation and modulation of neocortical spindle bursts. In vivo [16] and in vitro data [34] suggest that GABAergic synapses are not crucial for the generation of spindle bursts or spindle burst-like activity, respectively, but are essential for their spatial confinement to a cortical (pre-) column. In contrast, spindle bursts depend on intact glutamatergic synapses including alpha-amino-3-hydroxy-5-methyl-4-isoxazole propionic acid (AMPA) and N-methyl-D-aspartate (NMDA) receptors [34, 45]. Carbachol-induced spindle-like oscillations in P0–P3 mouse neocortex in vitro [34] and spindle bursts in P0–P2 rat in vivo [17] are blocked or significantly reduced by different gap junction blockers, indicating that electrical synapses are critically involved in the generation of spindle bursts at this neonatal period. However, Minlebaev et al. [16] reported for P1–P3 rats a significant increase in the occurrence of spontaneous spindle bursts in S1 following application of the gap-junction blocker mefloquine.

**Figure 6:**
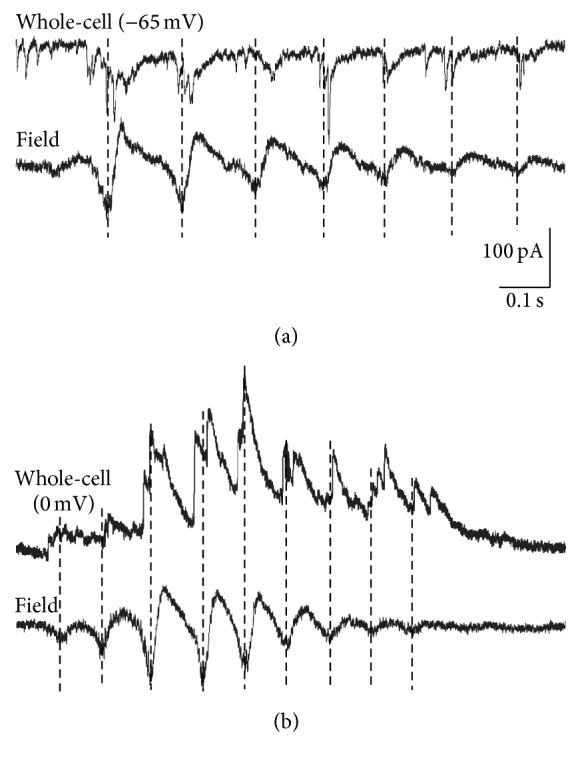
Synaptic events underlying a spindle burst recorded in S1 of a P6 rat in vivo. (a) Simultaneous registration of membrane currents (upper trace) and local field potential (lower trace). In this recording the membrane potential was held at −65 mV to isolate glutamatergic postsynaptic currents (PSCs). Note that the glutamatergic PSCs are phase locked to the field potential oscillations. (b) Recording at a holding potential of 0 mV to isolate GABAergic PSCs. Note that GABAergic PSCs are also phase locked to the field potential oscillations. Reproduced with permission from [14].

Single-cell recordings revealed additional insights into the mechanisms underlying the generation of spindle bursts. Spindle bursts in V1 [15] and S1 [14] are accompanied by a barrage of glutamatergic and GABAergic postsynaptic currents (PSCs) that are phase-locked to the spindle oscillations (Figure 6). In prefrontal cortex, glutamatergic and GABAergic synaptic inputs to excitatory pyramidal neurons are phase locked to the theta-band component of the spindle, while PSCs of inhibitory interneurons are phase locked to the higher beta and gamma frequencies, suggesting that excitatory and inhibitory neurons differentially modulate the distinct components of the spindle bursts [46]. Further, the application of CNQX eliminates the glutamatergic PSCs and completely blocks the occurrence of spindle bursts [16], indicating a major causal role of AMPA-receptor mediated glutamatergic inputs in the generation of spindle bursts. Additional insights have been obtained by experiments in which subplate cells were selectively ablated in S1 shortly after birth [36]. In these animals the deletion of subplate neurons caused a massive reduction of spindle burst activity. These in vivo results support previous in vitro studies, in which spindle bursts elicited by cholinergic stimulation were suppressed after removal of the subplate (see below) [34, 35]. Taken together, these data suggest that thalamocortical inputs—relayed and amplified by the subplate [28, 29]—play an important role in the generation of spindle bursts (e.g., [17, 45]).

Spindle bursts in the cerebral cortex of newborn rats can be elicited [34, 35] and modulated by cholinergic mechanisms [47]. In vitro, spindle burst-like oscillations can be reliably induced by activation of muscarinic acetylcholine receptors, predominantly of the m1 and m5 type [35]. In vivo, spindle bursts in V1 are decreased by ~50% following the application of the muscarinic receptor antagonist atropine. Furthermore, blockade of acetylcholine esterase with physostigmine or direct stimulation of the cholinergic basal forebrain nuclei augmented the occurrence V1 spindle bursts [47], indicating that the cholinergic system facilitates spindle burst activity in developing cerebral cortex.

## 4. Synchrony and Cerebral Networks Associated with Spindles

As discussed above, spatially confined spindle bursts in newborn rat cortex synchronize a local neuronal network resembling a neocortical (pre-) column (Figure 3). Beside these intra-areal synchronization, spindle bursts are also synchronized between different cortical regions within one hemisphere (intrahemispheric). As described above, a tight functional correlation in spontaneous and stimulus-evoked spindle burst activity exists between S1 and M1 cortex (Figure 5). Spindle bursts with similar properties as those in V1 and S1 have been also recorded in vivo in the prefrontal cortex of urethane-anesthetized rats older than P2 [24]. The same authors demonstrate that the hippocampus drives this early activity in the prefrontal cortex.

Beside this intrahemispheric synchronization between different cortical regions, spindle bursts also interact between both hemispheres (interhemispheric). In vivo simultaneous recordings of spontaneous activity in homotopic cortical areas in both hemispheres at the same stereotaxic coordinates and depth have demonstrated that the amount of interhemispheric synchronization in newborn rats is initially rather low and increases during the first postnatal week [17]. This interhemispheric communication of spindle burst activity depends on an intact corpus callosum. In this regard, in unanesthetized newborn rats callosotomy doubled the occurrence of spontaneous spindle bursts, suggesting that the corpus callosum modulates functionally inhibitory interactions between homotopic regions in both hemispheres during the occurrence of spindle burst activity [48]. Experiments in P2–P15 rats demonstrated that this callosotomy-induced disinhibition is a transient feature of early development that disappears abruptly after P6 [49].

It is not surprising that intra- and interhemispheric interactions of spontaneous activity in the spindle burst frequency range can be also observed in developing human cerebral cortex at early stages. Using EEG and functional magnetic resonance imaging (fMRI) Omidvarnia et al. could demonstrate in premature and full term human babies the existence of an “electric resting-state network” that shows functional intra- and interhemispheric interactions in the 8–15 Hz frequency range [50].

In summary, several in vitro and in vivo studies have demonstrated that spindle bursts represent elementary states of intra- and interhemispheric synchronization in the very immature cerebral cortex.

## 5. Physiological and Pathophysiological Role of Spindle-Like Oscillations in Early Brain Development

An increasing amount of experimental and clinical data strongly indicate that spindle bursts play an important role in the physiological development of the cerebral cortex. Experimental evidence indicates that spindle bursts may be particularly suited to interfere with early neurodevelopmental processes, and thus disturbances in spindle burst activity may cause long-term structural and functional disorders.

At early stages of development spontaneous and sensory-evoked activity patterns influence a variety of developmental processes, such as neurogenesis [51], apoptosis [52], neuronal migration [53], cellular differentiation [54], network formation [4], and myelination [55] (for review, see [3]). It is not completely understood how electrical activity controls these different developmental processes and whether distinct activity patterns, such as spindle bursts, play a specific role. However, for the control of apoptotic cell death of immature neurons in vitro and in vivo the essential role of spontaneous network bursts to provide antiapoptotic signals has been demonstrated [52, 56, 57].

For this activity-dependent control of neuronal survival the phosphatidylinositol 3-kinase pathway plays an important role, while the MAPK/extracellular signal-regulated kinase or the calcium/calmodulin-dependent protein kinase pathway is not directly involved [58]. Since one spontaneous spindle burst is associated at the single neuron level with 5–10 action potentials [16] and the frequency of spontaneous spindle bursts is ~5 per minute [16, 17], a single neuron discharges with 25–50 action potentials per minute. Under in vitro conditions this discharge frequency supports neuronal survival of developing neocortical neurons [56], suggesting that spontaneous spindle bursts in vivo provide an important physiological signal for the control of neuronal survival versus apoptosis in the neonatal cerebral cortex. Notably, spindle burst and gamma activity provides an ideal physiological stimulus for the activity-dependent release of BDNF, an important antiapoptotic signal [59]. Balkowiec and Katz [60] demonstrated for neuronal cultures that 30–60 min of electrical burst stimulation (50 pulses at 20–50 Hz at intervals of 20 s) increased extracellular BDNF levels by 20-fold, whereas stimulation patterns at lower frequency (albeit producing the same number of extracellular electric shocks) were ineffective (for review, see [61]). These data indicate that spontaneous spindle bursts represent a physiological trigger for the release of BDNF, which plays an important role in several aspects of development (for review, see [59]).

Using in vitro and in vivo models it has been recently shown that an experimentally induced inflammation by application of the endotoxin lipopolysaccharide induces rapid (<2 h) alterations in the pattern of spontaneous spindle bursts and gamma oscillations in neonatal rodent cerebral cortex, which subsequently leads to increased apoptotic cell death [57]. These inflammatory effects are specifically initiated by the microglia-derived proinflammatory cytokine tumor necrosis factor alpha and to a lesser extent by the chemokine macrophage inflammatory protein 2 [57]. Thus, inflammation causes a fast dysfunction in the pattern of spontaneous burst activity, which subsequently leads to increased apoptotic cell death, most likely by disturbances in the release of survival factors such as brain-derived neurotrophic factor (BDNF) acting on neurotrophin tropomyosin-related kinase B/C receptor [52]. Furthermore, removal of the subplate massively reduced spindle burst activity and led to a persistent loss of the typical barrel-like whisker representation within layer 4 [36], indicating that spindle bursts play a role in the development of the neocortical architecture.

In summary, these experimental data suggest that any disturbances in the spontaneous activity of the developing cerebral cortex (including spontaneous spindle bursts) induce acute dysfunctions, which may cause long-term disorders. However, it remains to be elucidated whether spindle burst in particular can be causally related to neurodevelopmental disturbances. It has been recently shown in extremely preterm infants that the properties of neocortical bursts recorded with EEG and their scaling relationships correlate significantly with later cognitive development [62]. These clinical data suggest that analyses of burst shapes obtained in EEG recordings from preterm and full-term newborn babies may have diagnostic use in neonatal intensive care units and predict the clinical outcome [63].

## 6. Open Questions

While it is generally accepted that early electrical activity shapes the maturation of neocortical circuits [3], it remains an open question whether the specific properties of spindle burst are required or fulfill a distinct role in development. In this regard, it is possible that the spatiotemporal patterns of spindle bursts translate into a local molecular signal which fulfills an important developmental role. In particular the role of GABAergic synaptic activity during spindle bursts is currently unclear. As GABA does not seem to be necessary for the generation or maintenance of spindle bursts, and since GABA is essential for neuronal differentiation [64, 65], it is tempting to speculate that spindle bursts control the spatially confined release of GABA in developing local networks.

Another unresolved issue is that both spindle bursts and gamma oscillations can be observed during the same period of early development. Intriguingly, both activity patterns share many features. They can be observed in early postnatal rodent brain from the day of birth ([17]; also see [66]) and occur spontaneously as well as after sensory stimulation. Moreover, both can be observed mainly during the critical periods of the primary sensory areas, although spindle bursts probably persist for slightly longer periods. In addition, spindle bursts and early gamma oscillations in newborn cortex are proposed to rely on thalamocortical inputs, in contrast to gamma oscillations in adult cortex which depend on perisomatic GABAergic inhibition [20]. Beside their differences in duration, occurrence, and frequency (see above), spindle bursts and gamma oscillations in newborn cortex reveal a number of additional distinctions. In the immature barrel cortex the vast majority (>90%) of spontaneously occurring spindle burst spans several barrel-related columns, whereas the majority (~70%) of spontaneous gamma oscillations are restricted to a single or two barrel related columns [17]. In line with these observations, gamma oscillations evoked by tactile stimulation are also closely related to a single functional column, while evoked spindle bursts span over more than one column [17]. Thus, one reason for the coexistence of spindle bursts and gamma oscillations during early postnatal development might be a distinct role in neocortical maturation. Gamma bursts may reflect local information processing mostly within a single functional column, thus supporting the maturation of a column related network. In contrast, spindle bursts probably reflect larger local network events and may thus serve to promote the connectivity between neighboring columns.

Although spindle bursts as well as gamma oscillations are spatially confined to a small network in one neocortical area, it is unclear to what extent and how this activity connects to other cortical and subcortical regions (e.g., see [18, 19]). Further, it remains to be studied whether the immature brain shows a spindle burst related “resting state” and how this network state is altered by sensory activation or by pathophysiological events.

Finally, it would be most interesting and important to correlate specific patterns of spontaneous activity (e.g., delta brush) recorded by means of full-band direct-current EEG in preterm und full-term human neonates with the acute functional state and with the further development of the child, as impressively done by Vanhatalo and colleagues [62, 67].

## 7. Conclusions

Spatially confined spindle bursts and delta brushes represent the most prominent physiological activity patterns in the developing cerebral cortex of newborn rodents and preterm human infants, respectively. Spontaneous and stimulus-evoked spindle bursts can be observed in various neocortical areas of different mammalian species and play important roles in the early development of cortical networks. However, it remains to be studied in more detail how exactly spindle bursts influence the maturation of the cortex and how a potential long-term dysfunction due to disturbances in spindle burst activity can be prevented by early intervention. Since this important type of brain activity is already present in the human fetus in utero (either spontaneously occurring or related to sensory inputs from the uterine environment), a better understanding of the physiological relevance of spindle burst oscillations is of major clinical relevance.
